# Heart failure, recurrent vascular events and death in patients with ischemic stroke—*results of the MonDAFIS study*

**DOI:** 10.1007/s11739-024-03594-8

**Published:** 2024-04-15

**Authors:** Serdar Tütüncü, Manuel C. Olma, Claudia Kunze, Joanna Dietzel, Johannes Schurig, Michael Rosenkranz, Robert Stingele, Matthias Maschke, Peter U. Heuschmann, Paulus Kirchhof, Ulrich Laufs, Darius G. Nabavi, Joachim Röther, Götz Thomalla, Roland Veltkamp, Matthias Endres, Karl Georg Haeusler, for the MonDAFIS Investigators

**Affiliations:** 1 Center for Stroke Research Berlin, Charité, Universitätsmedizin Berlin, Charitéplatz 1, 10117 Berlin, Germany; 2 Klinik und Hochschulambulanz für Neurologie mit Abteilung für Experimentelle Neurologie, Charité-Universitätsmedizin Berlin, Berlin, Germany; 3 Klinik für Neurologie und Neurologische Frührehabilitation, Albertinen-Krankenhaus Hamburg, Hamburg, Germany; 4 Department of Neurology, German Red Cross Hospital Berlin Köpenick, Berlin, Germany; 5 Krankenhaus der Barmherzigen Brüder Trier, Klinik für Neurologie und Neurophysiologie, Trier, Germany; 6 Institute of Clinical Epidemiology and Biometry, University Würzburg, Würzburg, Germany; 7 Comprehensive Heart Failure Center & Clinical Trial Centre Würzburg, University Hospital Würzburg, Würzburg, Germany; 8 German Atrial Fibrillation Network (AFNET), Münster, Germany; 9 Institute of Cardiovascular Sciences, College of Medical and Dental Sciences, Medical School, University of Birmingham, Birmingham, UK; 10 Department of Cardiology, UHB and SWBH NHS Trusts, Birmingham, UK; 11 University Heart and Vascular Center Hamburg, Hamburg, Germany; 12 Klinik und Poliklinik für Kardiologie, Universitätsklinikum Leipzig, Leipzig, Germany; 13 Department of Neurology, Vivantes Klinikum Neukölln, Berlin, Germany; 14 Department of Neurology, Asklepios Klinik Altona, Hamburg, Germany; 15 Department of Neurology, University Medical Center Hamburg-Eppendorf, Hamburg, Germany; 16 Department of Neurology, Alfried Krupp Krankenhaus, Essen, Germany; 17 Department of Brain Sciences, Imperial College London, London, UK; 18 German Center for Neurodegenerative Diseases, Partner Site Berlin, Berlin, Germany; 19 German Center for Cardiovascular Diseases, Partner Site Berlin, Berlin, Germany; 20 Excellence Cluster NeuroCure, Berlin, Germany; 21 Berlin Institute of Health (BIH), Berlin, Germany; 22 Department of Neurology, Universitätsklinikum Würzburg, Würzburg, Germany

**Keywords:** Ischemic Stroke, Heart Failure, Vascular Outcome, Mortality

## Abstract

Heart failure (HF) is associated with poor outcome after stroke, but data from large prospective trials are sparse.

We assessed the impact of HF on clinical endpoints in patients hospitalized with acute ischemic stroke or transient ischemic attack (TIA) enrolled in the prospective, multicenter *Systematic Monitoring for Detection of Atrial Fibrillation in Patients with Acute Ischemic Stroke* (MonDAFIS) trial. HF was defined as left ventricular ejection fraction (LVEF) < 55% or a history of HF on admission. The composite of recurrent stroke, major bleeding, myocardial infarction, and all-cause death, and its components during the subsequent 24 months were assessed. We used estimated hazard ratios in confounder-adjusted models. Overall, 410/2562 (16.0%) stroke patients fulfilled the HF criteria (i.e. 381 [14.9%] with LVEF < 55% and 29 [1.9%] based on medical history). Patients with HF had more often diabetes, coronary and peripheral arterial disease and presented with more severe strokes on admission. HF at baseline correlated with myocardial infarction (HR 2.21; 95% CI 1.02–4.79), and all-cause death (HR 1.67; 95% CI 1.12–2.50), but not with major bleed (HR 1.93; 95% CI 0.73–5.06) or recurrent stroke/TIA (HR 1.08; 95% CI 0.75–1.57). The data were adjusted for age, stroke severity, cardiovascular risk factors, and randomization. Patients with ischemic stroke or TIA and comorbid HF have a higher risk of myocardial infarction and death compared with non-HF patients whereas the risk of recurrent stroke or major hemorrhage was similar. *Trial registration number* Clinicaltrials.gov NCT02204267.

**Supplementary Information:**

The online version contains supplementary material available at 10.1007/s11739-024-03594-8.

## Introduction

The prevalence of both stroke and heart failure (HF) is high in the elderly, and there is a long list of common cardiovascular risk factors, including hypertension, diabetes, sleep apnea, kidney dysfunction, or atrial fibrillation (AF). HF is regarded as an independent risk factor for ischemic stroke, and about 9% of all ischemic strokes are assumed to be related to HF [1]. Furthermore, there is an association between HF with unfavorable clinical outcome and mortality after stroke [2–6]. In addition, a reduced ejection fraction in patients with ischemic stroke and atrial fibrillation is associated with mortality [7]. In contrast, it is less clear whether HF is also associated with recurrent vascular events following an ischemic stroke. Data on recurrent ischemic stroke in stroke patients with HF are conflicting: a systemic review and meta-analysis of seven prospective trials with differing follow-up times demonstrated a significant association of HF with recurrent ischemic stroke in 9173 ischemic stroke patients [8]. However, a retrospective insurance data-based analysis showed no significant association of recurrent stroke and HF in 370,527 ischemic stroke patients [9]. While there are several publications focusing on myocardial infarction following ischemic stroke [10, 11], data on the association of myocardial infarction with HF in ischemic stroke patients are scarce [12]. In addition, studies examining the occurrence of major bleeding in association with heart failure in ischemic stroke patients are missing.

In this post-hoc analysis of the prospective multicenter MonDAFIS study, we analyzed the impact of HF at baseline on the composite of recurrent stroke, major bleeding, myocardial infarction, and all-cause death within 24 months after hospitalization for acute ischemic stroke or transient ischemic attack (TIA) [13, 14]. Furthermore, we investigated the association of each vascular endpoint separately with HF and rates of oral anticoagulation after 24 months in patients with or without HF at baseline.

## Methods

### Study cohort

MonDAFIS was an investigator-initiated randomized trial sponsored by the Charité—Universitätsmedizin Berlin, Berlin, Germany, and supported by an unrestricted research grant from Bayer Vital GmbH, Leverkusen, Germany to the Charité. The study rationale and design [12] as well as the primary and secondary endpoints [14] were published previously. The MonDAFIS study received primary approval from the Ethics Committee of the Charité—Universitätsmedizin Berlin, Germany. All 39 participating study centers provided approval from their respective ethics committees. All study patients gave written informed consent. The MonDAFIS trial complies with the Declaration of Helsinki. Patients were recruited from December, 2014 to September, 2017. A critical event committee adjudicated all serious adverse events (including recurrent stroke, myocardial infarction, major bleeding, and all-cause death). The members met at the study coordinating center and were blinded to trial randomization.

### Study population

Men or women ≥ 18 years of age were eligible for study enrollment if they had an ischemic stroke according to WHO criteria [15]. If the newly occurring neurological deficits were transient in nature (consistent with a TIA), an additional inclusion criterion required either a neurologist to have observed and documented the acute neurological deficits or evidence of a corresponding acute ischemic lesion on imaging. Patients were excluded due to withdrawal of informed consent and data deletion or lack of any data. Detailed information regarding further in- and exclusion criteria as well as the trial intervention was published previously [14].

In the vast majority of MonDAFIS patients, left ventricular ejection fraction (LVEF) was determined by echocardiography at baseline as part of routine diagnostic procedures. The LVEF was captured in the eCRF as a categorized variable (LVEF ≥ 55% (“normal”), LVEF 31–54% (“slightly reduced”), LVEF ≤ 30% (“reduced”)). The rationale for this definition was based on the ESC guidelines for measuring LVEF that was valid at the time of study conception. In addition, heart failure (HF) was considered as pre-existing at baseline if it was reported by the patient or evident from existing medical reports (history of HF). In the present analysis, patients with a documented LVEF < 55% and/or a history of HF at baseline were considered HF patients. Patients without echocardiography during the index-stroke/TIA related hospital stay were excluded from the present analysis.

### Outcomes

In line with the pre-defined secondary endpoint of the MonDAFIS study, we assessed the composite of recurrent stroke, myocardial infarction, major bleed, and all-cause death within 2 years after the index stroke in defined subgroups. Furthermore, the individual components of the composite endpoint were analyzed separately. The rate of oral anticoagulation within 24 months after the index event was assessed in patients with and without HF at baseline.

### Statistical methods

This is an explorative analysis of the MonDAFIS study data set using predefined outcomes. Baseline characteristics are reported as frequencies and percentages for categorical variables or median and limits of the interquartile range (IQR; [25th and 75th percentile]) for metric variables. For the outcomes of interest, we conducted event-free survival analyses comparing cumulative hazards between patient groups. Event-free survival time, as well as survival, was measured in person-days until one of the events of the combined endpoint or death occurred, the study ended, or the participant was lost to follow-up. These dropouts are censored at the time of last contact. We used Kaplan–Meier curves and the log-rank test to compare crude cumulative hazard distributions. Cox Proportional Hazard models (crude and adjusted for age and cardivascular risk factors as arterial hypertension, diabetes mellitus, hyperlipoproteinemia, coronary heart disease, detection of atrial fibrillation and group randomization as well as stroke severity measured by the NIHSS score at baseline given as model 1 and model 2 with further adjustment for intravenous thrombolysis and endovascular thrombectomy in addition) were used to estimate hazard ratios (HR) for each vascular endpoint, the composite endpoint for all-cause death risk within 2 years after ischemic stroke or TIA. In a further sensitivity analysis, we added the status of antithrombotic therapy (antiplatelets or oral anticoagulation) at discharge after the index stroke in to the above-mentioned models. Cox Proportional Hazard assumption was checked. For the endpoint ‘oral anticoagulation 24 months after index event’ a multiple binary logistic regression analysis (adjusted for AF and detection of left atrial and/or ventricular thrombus) was conducted and adjusted odds ratios, as well as respective confidence intervals (CI) are reported. Data preparation was done using the software IBM SPSS Statistics 24.

## Results

**Fig. 1:**
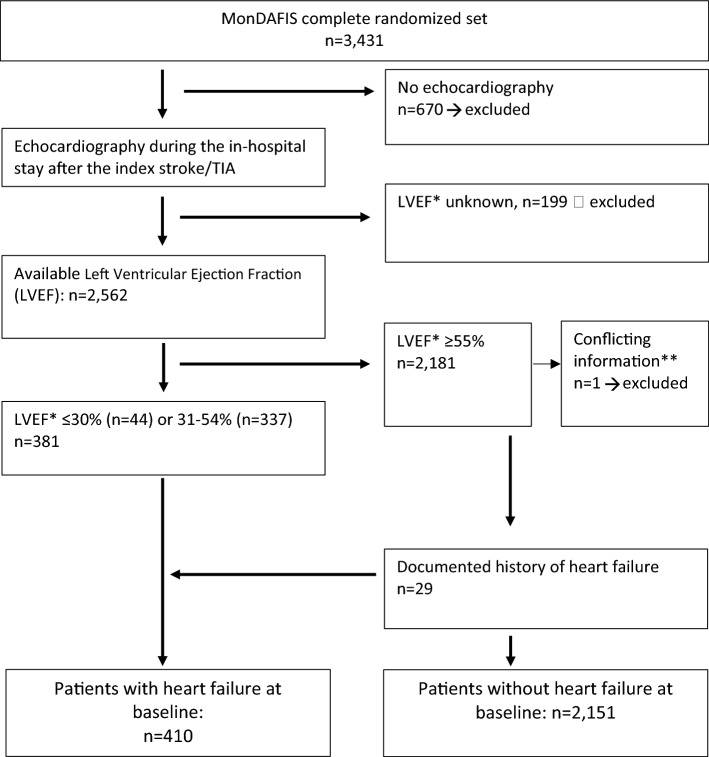
Derivation of the study population. *LVEF left ventricular ejection fraction, **Data of one MonDAFIS patient were excluded from analysis due to conflicting information regarding first diagnosis of heart failure during in-hospital stay

Of 3,431 patients included in MonDAFIS, LVEF was available in 2562 (75%) patients (mean age 67 [57–76] years, 39.5% female, median NIHSS score on admission 2 points [1–4]). Figure 1 shows the derivation of the study population. Patients without documented LVEF were older, more often had a TIA as index event and a stroke or TIA before the index event (Table 1 Online Supplement).

Overall, 381/2562 (14.9%) patients had a LVEF < 55% and 75/2562 patients had a documented past medical history of HF (of which 29 had a LVEF ≥ 55%). Therefore, a total of 410/2562 (16.0%) patients fulfilled at least one of the pre-defined HF criteria (Fig. 1). Baseline characteristics and results of multiple binary logistic regression of patients with vs without HF are shown in Table 1.

**Table 1:** Baseline characteristics of MonDAFIS study patients with or without heart failure at baseline

	Univariate analysis		*p*-value	Multivariate analysis	
	Heart failure, *n* = 410	No heart failure, *n* = 2151		OR (95% CI)*	*p*-value
Age, years, median [IQR]	69 [60–77]	66 [56–76]	< 0.001	1.01 (0.99–1.02)	0.078
Age categories, years, *n* (%)			0.001	-	-
< 65	149 (36.3)	991 (46.1)		-	-
65–74	121 (29.5)	566 (26.3)		-	-
75–84	116 (28.3)	521 (24.2)		-	-
> 84	24 (5.9)	73 (3.4)		-	-
Female sex, *n* (%)	119 (29.0)	866 (40.3)	< 0.001	0.79 (0.61–1.03)	0.081
Body weight, kg, mean (SD) [IQR]	81 [70–95]	80 [70–90]	0.009	1.01 (1.00–1.02)	0.052
Index stroke, * n* (%)			0.020		
TIA	99 (24.1)	640 (29.8)		1	
S troke	311 (75.9)	1507 (70.2)		1.11 (0.86–1.44)	0.422
NIHSS score on admission, median [IQR]	3 [1–5]	2 [1–4]	0.013	1.05 (1.02–1.09)	0.002
In-hospital treatment, *n* (%) Intravenous thrombolysis	91 (22.2)	500 (23.2)	0.644	-	-
Endovascular treatment	15 (3.7)	56 (2.6)	0.233	-	-
Hemicraniectomy	1 (0.2)	1 (0.0)	0.190	-	-
Carotid surgery or stenting	9 (2.2)	33 (1.5)	0.334	-	-
Cardiovascular risk factors, *n* (%)					
Diabetes mellitus	150 (36.6)	508 (23.6)	< 0.001	1.33 (1.04–1.70)	0.026
Hypertension	346 (84.4)	1,615 (75.2)	< 0.001	1.14 (0.83–1.57)	0.408
Hypercholesterolemia	237 (57.8)	1,125 (52.4)	0.043	1.06 (0.84–1.33)	0.634
Coronary heart disease	116 (28.3)	191 (8.9)	< 0.001	3.12 (2.36–4.13)	< 0.001
Peripheral arterial disease	34 (8.3)	60 (2.8)	< 0.001	2.04 (1.28–3.25)	0.003
Prior ischemic stroke or TIA	68 (16.6)	326 (15.2)	0.469	-	-
Smoker	211 (51.7)	1017 (47.6)	0.129	-	-
Randomization to the intervention group, *n* (%)	200 (48.8)	1072 (49.8)	0.695	-	-

^**^OR and 95% CI for continues variables were expressed per point (BMI, NIHSS) or per each year of age

Patients with HF more often had diabetes mellitus [OR 1.33; 95% CI 1.04–1.70; *P* = 0.026], coronary artery disease [OR 3.12; 95% CI 2.36–4.13; *P* < 0.001], and peripheral arterial disease [OR 2.03; 95% CI 1.27–3.24; *P* = 0.003] than non-HF patients. Moreover, patients with HF had a higher stroke severity on admission than patients without HF [assessed by the NIHSS score; OR per point 1.05; 95% CI 1.02–1.09; *P* = 0.002).

### Association of heart failure with clinical outcomes

Within two years after index stroke or TIA, the predefined composite endpoint occurred in 480/2561 (14.0%) of patients, including recurrent stroke in 301 (8.8%) patients, myocardial infarction in 43 (1.3%) patients, major bleeding in 32 (0.9%) patients and all-cause death in 175 (5.1%) patients. Recurrent ischemic stroke or TIA occurred in 283/2561 (8.3%) patients, while 18/2561 (0.7%) patients had a hemorrhagic stroke within 2 years.

**Fig. 2:**
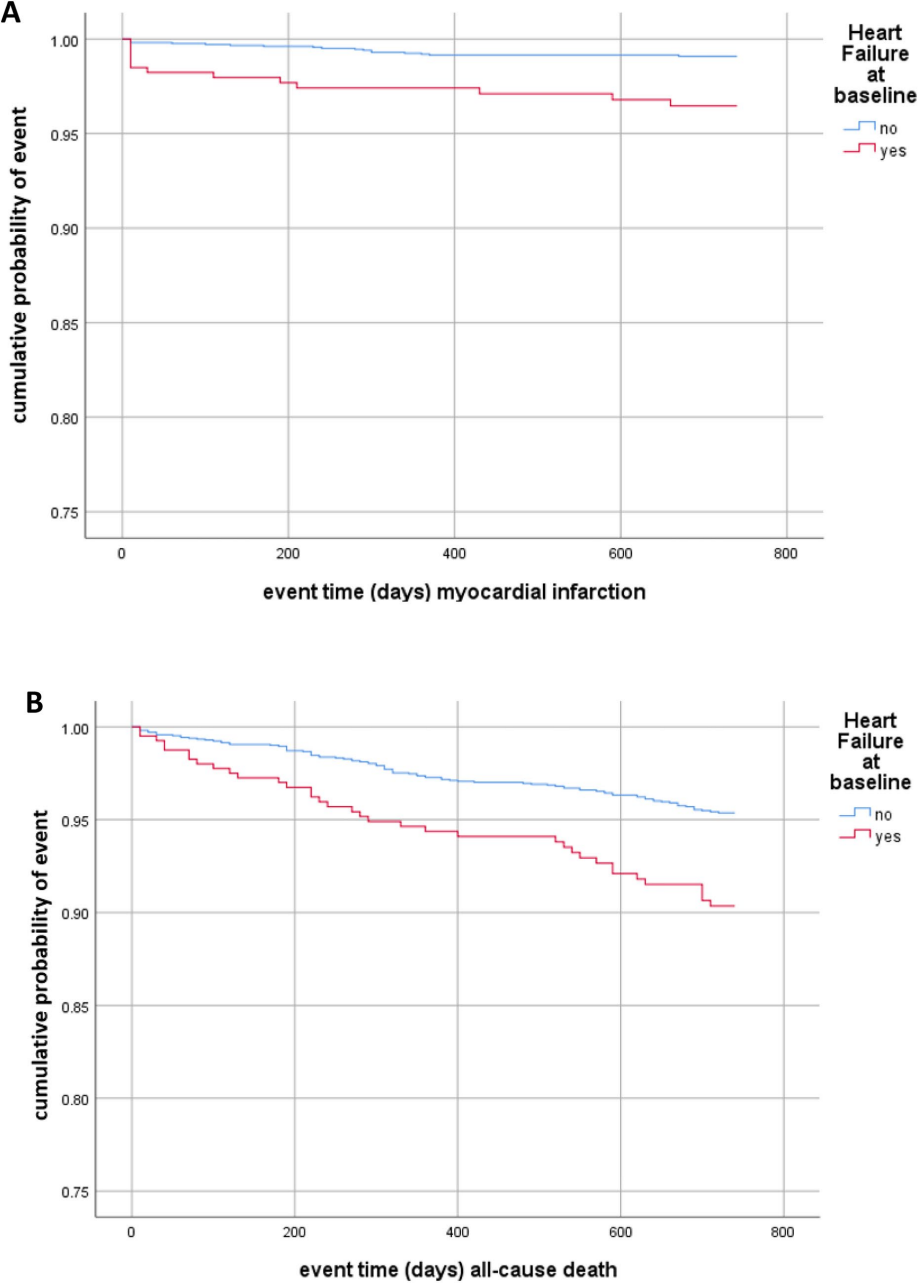
Time to event analysis. Occurrence of myocardial infarction (**A**) or all-cause death (**B**) within 24 months according to heart failure status at baseline

After adjusting for cardiovascular risk factors, stroke severity and randomization (model 1), HF was associated with myocardial infarction [HR 2.21; 95% CI 1.02–4.79; *P* = 0.046], and all-cause death [HR 1.67; 95% CI 1.12–2.49, *P* = 0.013] within 2 years after the index event. The event rates at different time frames for myocardial infarction and all-cause death are shown in Tables 2 and 3 Online Supplement. On the contrary, neither the composite endpoint [HR 1.28; 95% CI 0.99–1.66] nor recurrent ischemic stroke or TIA [HR 1.07; 95% CI: 0.74–1.55] nor major bleeding [HR 1.93; 95% CI 0.73–5.06] (Fig. 2 and Table 2) was significantly increased in stroke patients with HF. Additional adjustment for intravenous thrombolysis and endovascular thrombectomy (model 2) yielded similar results (Table 2). Also, excluding patients with a documented medical history of HF but normal LVEF (≥ 55%) at baseline yielded similar results (Table 4 Online Supplement). Adding the status of 'no-antithrombotic’ treatment at hospital discharge after the index stroke into both models of the cox regression analysis did not change the significant impact of heart failure in the occurrence of myocardial infarction or all-cause death.

**Table 2:** Adjusted survival analyses, Cox regression, hazard ratios for the composite endpoint (recurrent stroke, myocardial infarction, major bleeding, all-cause death) and its components as well as recurrent ischemic stroke/TIA within 24 months after the index stroke/TIA for patients with heart failure (*n* = 410) compared to those without heart failure at baseline

	Crude		Adjusted, Model 1^a^		Adjusted, Model 2^a^	
	HR (95% CI)	*P*	HR (95% CI)	*P*	HR (95% CI)	*P*
Composite endpoint^b^	1.54 (1.20–1.98)	0.001	1.28 (0.99–1.66)	0.065	1.29 (0.99–1.67)	0.060
All-cause death	2.15 (1.46–3.16)	< 0.001	1.67 (1.12–2.49)	0.013	1.65 (1.10–2.47)	0.015
Recurrent stroke^c^	1.21 (0.86–1.69)	0.274	1.08 (0.76–1.54)	0.668	1.09 (0.77–1.56)	0.620
Recurrent ischemic stroke or TIA	1.16 (0.81–1.65)	0.425	1.07 (0.74–1.55)	0.737	1.08 (0.75–1.57)	0.680
Recurrent ischemic stroke	1.24 (0.83–1.85)	0.299	1.08 (0.71–1.66)	0.719	1.09 (0.71–1.67)	0.683
Recurrent TIA	0.91 (0.43–1.93)	0.806	0.98 (0.46–2.11)	0.955	0.99 (0.46–2.14)	0.986
Myocardial infarction	3.96 (1.94–8.08)	< 0.001	2.21 (1.02–4.79)	0.046	2. 18 (1.01–4.76)	0.050
Major bleed	1.93 (0.76–4.89)	0.168	1.93 (0.73–5.06)	0.184	1.89 (0.72–4.99)	0.196

^a^Adjusted Model 1: for age, stroke severity (NIHSS score on admission), diabetes mellitus, arterial hypertension, hyperlipoproteinemia, coronary heart disease, detection of atrial fibrillation in-hospital, randomization; Adjusted Model 2: all variables of Adjusted Model 1 and additionally intravenous thrombolysis and endovascular thrombectomy

^b^Recurrent stroke, MI, major bleed, all cause death c) Ischemic Stroke, Transient Ischemic Attack, Intracerebral Hemorrhage, Subarachnoid Hemorrhage

### Secondary stroke prevention in patients with heart failure

Information on oral anticoagulation at 24 months was available in 2072/2561 (80.9%) patients and, similarly, on antiplatelet treatment in 2075/2561 (81.0%) patients. Overall, 312/2,072 (15.1%) patients were on anticoagulation and 1,645/2,075 (79.4%) patients on antiplatelets at 24 months. In patients with HF at baseline, information on oral anticoagulation at 24 months was available in 304/410 (74.2%) patients. There was a statistically significant difference in the rate oral anticoagulation in HF patients vs. non-HF patients (63/304 [20.7%] vs. 249/1768 [14.1%], respectively, *P* = 0.004). AF was diagnosed in 46/63 (73.0%) of those with oral anticoagulants and HF. The rate of oral anticoagulation was 15.7% in HF patients vs 11.8% in non-HF patients at 6 months after the index stroke/TIA (p = 0.046) and 16.6% vs 12.9% after 12 months (*P* = 0.072) (Table 5 online supplement).

After multivariate adjustment (including newly detected AF within 24 months and left ventricular or left atrial thrombus at baseline), there was no significant association between HF status at baseline and oral anticoagulation after 24 months [OR 0.89; 95% CI 0.51–1.56; *P* = 0.689].

## Discussion

The prospective multicenter MonDAFIS study shows that ischemic stroke or TIA patients with comorbid HF had a significantly higher rate of myocardial infarction and all-cause mortality within 24 months than non-HF patients. One strength of our post hoc analysis is the large number of clinical endpoints assessed by an independent endpoint committee and standardized follow-up for 24 months. Our data confirm that stroke/TIA patients with comorbid HF are not only more likely to have cardiovascular risk factors (such as diabetes, coronary heart disease, peripheral arterial disease) but also to have more severe strokes than stroke patients without HF [2]. Our finding of a statistically higher rate of myocardial infarction over 24 months after stroke/TIA in the presence of HF is consistent with the results of a large multicenter registry in the UK based on NHS primary care data of 9,840 patients [12]. The detected higher mortality rate in ischemic stroke patients with comorbid HF is also in line with previous studies [6, 9]. Of note, the rate of recurrent stroke as well as recurrent ischemic stroke or TIA was not associated with HF in our study, which was also found in a retrospective analysis of a large registry but is not consistent with a meta-analysis of prospective studies [8, 9]. The follow-up period of 2 years was may be too short to observe any potential association between HF and ischemic stroke—the authors of the WARCEF trial reported a higher rate of ischemic strokes during a follow-up period that was approximately 1.5 years longer. One study reported a significant association of recurrent intracerebral hemorrhage in patients with HF compared to non-HF patients [9]. In MonDAFIS, the HF status at baseline was not associated with the rate of major bleeding after stroke, even though 21% of HF patients received oral anticoagulation at 24 months. In fact, OAC status was not statistically different between HF vs. non-HF patients after adjusting for AF and presence of cardiac thrombi.

There are some limitations that should be mentioned. First, the MonDAFIS study was not designed to investigate the impact of HF on recurrent vascular events or death. Second, as echocardiography was conducted according to routine clinical practice respective data were available for only 75% of the total study population. Third, we had no systematic information on biomarkers, like natriuretic peptides. Fourth, we had no further information on HF status after hospital discharge after the index-stroke/TIA. Fifth, our results cannot be applied to all stroke patients, as patients had to provide informed consent to participate in the MonDAFIS study. Finally, we are not able to specify the cause of death. Due to formal data protection laws we did not have access to death certificates and autopsy is not regularly performed even if patients die in hospital.

## Conclusion

Patients with acute ischemic stroke or TIA with comorbid HF have a higher prevalence of cardiovascular risk factors, suffered more severe (index) strokes and have a higher risk for myocardial infarctions or all-cause death within 24 months after the index stroke. Special attention should be paid to the prevention of myocardial infarction in this patient population and cardiac follow-up should be recommended in stroke patients with HF.

### Supplementary Information

Below is the link to the electronic supplementary material.

Supplementary file1 (DOCX 26 KB)

## Data Availability

Due to data protection regulations, we cannot release the data, as the patient consent form specifically defined or limited data sharing to the evaluating biometric center. Therefore, upon request, it can be decided whether a transfer to third parties can be facilitated through an ethics approval and data protection approval from the Charité.

## References

[1] Doehner W, Ural D, Haeusler KG et al (2018) Heart and brain interaction in patients with heart failure: overview and proposal for a taxonomy. A position paper from the Study Group on Heart and Brain Interaction of the Heart Failure Association. Eur J Heart Fail 20(2):199–215 29280256 10.1002/ejhf.1100

[2] Barkhudaryan A, Doehner W, Scherbakov N (2021) Ischemic stroke and heart failure: facts and numbers. An update. J Clin Med 10(5):1146 33803423 10.3390/jcm10051146 PMC7967189

[3] Haeusler KG, Laufs U, Endres M (2011) Chronic heart failure and ischemic stroke. Stroke 42(10):2977–2982 21903953 10.1161/STROKEAHA.111.628479

[4] Tai YH, Chang CC, Yeh CC et al (2020) Long-term risk of stroke and poststroke outcomes in patients with heart failure: two nationwide studies. Clin Epidemiol 12:1235–1244 33177880 10.2147/CLEP.S261179 PMC7652062

[5] Sennfalt S, Pihlsgard M, Petersson J, Norrving B, Ullberg T (2020) Long-term outcome after ischemic stroke in relation to comorbidity—an observational study from the Swedish Stroke Register (Riksstroke). Eur Stroke J 5(1):36–46 32232168 10.1177/2396987319883154 PMC7092731

[6] Scherbakov N, Haeusler KG, Doehner W (2015) Ischemic stroke and heart failure: facts and numbers. ESC Heart Fail 2(1):1–4 28834645 10.1002/ehf2.12026 PMC5746959

[7] RAF and RENO-EXTEND Investigators (2023) The risk of stroke recurrence in patients with atrial fibrillation and reduced ejection fraction. Eur Stroke J 8(3):731–737 37249094 10.1177/23969873231177625 PMC10472961

[8] Katsanos AH, Parissis J, Frogoudaki A et al (2016) Heart failure and the risk of ischemic stroke recurrence: a systematic review and meta-analysis. J Neurol Sci 362:182–187 26944144 10.1016/j.jns.2016.01.053

[9] Pana TA, Wood AD, Perdomo-Lampignano JA et al (2019) Impact of heart failure on stroke mortality and recurrence. Heart Asia 11(1):e011139 31244914 10.1136/heartasia-2018-011139 PMC6560925

[10] Alqahtani F, Aljohani S, Tarabishy A, Busu T, Adcock A, Alkhouli M (2017) Incidence and outcomes of myocardial infarction in patients admitted with acute ischemic stroke. Stroke 48(11):2931–2938 29018137 10.1161/STROKEAHA.117.018408

[11] Scheitz JF, Nolte CH, Doehner W, Hachinski V, Endres M (2018) Stroke-heart syndrome: clinical presentation and underlying mechanisms. Lancet Neurol 17(12):1109–1120 30509695 10.1016/S1474-4422(18)30336-3

[12] Pana TA, Wood AD, Mamas MA et al (2019) Myocardial infarction after acute ischaemic stroke: Incidence, mortality and risk factors. Acta Neurol Scand 140(3):219–228 31140583 10.1111/ane.13135

[13] Haeusler KG, Kirchhof P, Heuschmann PU et al (2016) Impact of standardized MONitoring for Detection of Atrial Fibrillation in Ischemic Stroke (MonDAFIS): rationale and design of a prospective randomized multicenter study. Am Heart J 172:19–25 26856211 10.1016/j.ahj.2015.10.010

[14] Haeusler KG, Kirchhof P, Kunze C et al (2021) Systematic monitoring for detection of atrial fibrillation in patients with acute ischaemic stroke (MonDAFIS): a randomised, open-label, multicentre study. Lancet Neurol 20(6):426–436 34022169 10.1016/S1474-4422(21)00067-3

[15] Hatano S (1976) Experience from a multicentre stroke register: a preliminary report. Bull World Health Organ 54(5):541–553 1088404 PMC2366492

