# Supplementary material for: Heart failure, recurrent vascular events and death in patients with ischemic stroke—results of the MonDAFIS study
Source: Intern Emerg Med. 2024 Apr 15;19(5):1247–54. doi: 10.1007/s11739-024-03594-8 (PMC11364684; doi:10.1007/s11739-024-03594-8)
Supplement: Supplementary file 1 — Supplementary file1 (DOCX 26 KB) [file 11739_2024_3594_MOESM1_ESM.docx]

**SUPPLEMANTARY INFORMATION**

**Heart Failure, Recurrent Vascular Events and Death in Patients with Ischemic Stroke - *Results of the MonDAFIS study***

Serdar Tütüncü, MD^1^ ; Manuel C. Olma, MD^1,2^ ; Claudia Kunze, BA^1^; Joanna Dietzel, MD^1^; Johannes Schurig, MD^1^; Michael Rosenkranz, MD^3^, Robert Stingele, MD^4^; Matthias Maschke, MD^5^, Peter U. Heuschmann, MD^6,7^; Paulus Kirchhof, MD^8-10^; Ulrich Laufs, MD^11^; Darius G. Nabavi, MD^12^; Joachim Röther, MD^13^; Götz Thomalla, MD^14^; Roland Veltkamp, MD^15,16^; Matthias Endres, MD^1,2,17-20*^; Karl Georg Haeusler, MD^21*^ for the MonDAFIS Investigators

^1^ Center for Stroke Research Berlin, Charité - Universitätsmedizin Berlin, Germany

^2^ Klinik und Hochschulambulanz für Neurologie mit Abteilung für Experimentelle Neurologie, Charité-Universitätsmedizin Berlin, Berlin, Germany

^3^ Klinik für Neurologie und Neurologische Frührehabilitation - Albertinen-Krankenhaus Hamburg

^4^ Department of Neurology, German Red Cross Hospital Berlin Köpenick, Germany

^5^ Krankenhaus der Barmherzigen Brüder Trier - Klinik für Neurologie und Neurophysiologie

^6^ Institute of Clinical Epidemiology and Biometry, University Würzburg, Germany

^7^ Comprehensive Heart Failure Center & Clinical Trial Centre Würzburg, University Hospital Würzburg, Germany

^8^ German Atrial Fibrillation Network (AFNET), Münster, Germany

^9^ Institute of Cardiovascular Sciences, College of Medical and Dental Sciences, Medical School, University of Birmingham, UK, and Departments of Cardiology, UHB and SWBH NHS Trusts, Birmingham, UK

^10^ University Heart and Vascular Center Hamburg, Hamburg, Germany

^11^ Klinik und Poliklinik für Kardiologie, Universitätsklinikum Leipzig, Leipzig, Germany

^12^ Department of Neurology, Vivantes Klinikum Neukölln, Berlin, Germany

^13^ Department of Neurology, Asklepios Klinik Altona, Hamburg, Germany

^14^ Department of Neurology, University Medical Center Hamburg-Eppendorf, Germany

^15^ Department of Neurology, Alfried Krupp Krankenhaus, Essen, Germany

^16^ Department of Brain Sciences, Imperial College London, UK

^17^ German Center for Neurodegenerative Diseases, Partner Site Berlin, Germany

^18^ German Center for Cardiovascular Diseases, Partner Site Berlin, Germany

^19^ Excellence Cluster NeuroCure, Berlin, Germany

^20^ Berlin Institute of Health (BIH), Berlin, Germany

^21^ Department of Neurology, Universitätsklinikum Würzburg, Germany

* Contributed equally

**Corresponding author:** Serdar Tütüncü, MD

Center for Stroke Research Berlin

Charitéplatz 1

10117 Berlin

Germany

Phone +4930450560687

Serdar.Tuetuencue@charite.de

| **Table 1 ONLINE SUPPLEMENT:** Baseline characteristics of MonDAFIS study patients included or not included into this subanalysis of the MonDAFIS study. | | | | | |
| --- | --- | --- | --- | --- | --- |
|  | **Univariate analysis** | |  | **Multivariate analysis** | |
|  | **Included patients**  n=2,561 | **Excluded patients**  n=868 | ***p-value*** | **OR (95% CI)*** | **p-value** |
| **Age, years,** median [IQR] | 66 [56-76] | 71 [60-78] | <0.001 | 0.98 (0.98-0.99) | <0.001 |
| **Age categories, years**, n (%) |  |  | <0.001 | - | - |
| <65 | 1140 (44.5) | 319 (36.8) |  | - | - |
| 65-74 | 687 (29.8) | 205 (23.6) |  | - | - |
| 75-84 | 637 (24.9) | 271 (31.2) |  | - | - |
| >84 | 97 (3.8) | 73 (8.4) |  | - | - |
| **Female sex**, n (%) | 985 (38.5) | 371 (42.7) | 0.030 | 0.89 (0.75-1.07) | 0.225 |
| **Weight, kg**, mean [IQR] | 80 [70-91] | 78 [68-90] | <0.001 | 1.00 (1.00-1.01) | 0.233 |
| **Index stroke**, n (%) |  |  | 0.009 |  |  |
| TIA | 739 (28.9) | 290 (33.7) |  | 1 |  |
| Stroke | 1818 (71.1) | 571 (66.3) |  | 1.26 (1.06-1.50) | 0.010 |
| **NIHSS score on admission**, median [IQR] | 2 [1-4] | 2 [1-4] | 0.004 | 1.02 (0.99-1.06) | 0.134 |
| **In-hospital treatment**, n (%)  Intravenous thrombolysis | 591 (23.1) | 154 (17.9) | 0.001 | 1.29 (1.04-1.62) | 0.024 |
| Endovascular treatment | 71 (2.8) | 27 (3.2) | 0.478 | - | - |
| Hemicraniectomy | 2 (0.1) | 2 (0.2) | 0.256 | - | - |
| Carotid surgery or stenting | 42 (1.6) | 36 (4.3) | <0.001 | 0.32 (0.20-0.52) | <0.001 |
| **Cardiovascular risk factors**, n (%) |  |  |  |  |  |
| Diabetes mellitus | 658 (25.7) | 223 (26.7) | 0.586 | - | - |
| Hypertension | 1961 (76.7) | 646 (77.5) | 0.671 | - | - |
| Hypercholesterolemia | 1362 (53.2) | 436 (52.3) | 0.660 | - | - |
| Coronary heart disease | 307 (12.0) | 106 (12.7) | 0.584 | - | - |
| Atrial fibrillation (newly detected) | 131 (5.1) | 34 (3.9) | 0.169 | - | - |
| Peripheral arterial disease | 94 (3.7) | 39 (1.2) | 0.217 | - | - |
| Prior ischemic stroke or TIA | 394 (15.4) | 194 (23.2) | <0.001 | 0.65 (0.53-0.79) | <0.001 |
| Smoker | 1228 (48.3) | 445 (51.9) | 0.069 | - | - |
| **Randomization to the intervention group**, n (%) | 1272 (49.7) | 441 (50.7) | 0.583 | - | - |

**OR and 95% CI for continues variables were expressed per point (BMI, NIHSS) or per each year of age.

| **Table 2 ONLINE SUPPLEMENT:** Rates of myocardial infarction according to heart failure at baseline during follow-up. | | | | | | |
| --- | --- | --- | --- | --- | --- | --- |
|  |  | **0-30 days** | **31-180 days** | **181-365 days** | **366-730 days** | **In total** |
| **Heart failure at baseline** | **Yes,** n=410 | 1.7%  (n=7) | 0.2 %  (n=1) | 0.5%  (n=2) | 0.7%  (n=3) | 3.2% (n=13) |
|  | **No,** n=2561 | 0.2%  (n=4) | 0.2%  (n=4) | 0.3%  (n=8) | 0.1%  (n=2) | 0.7% (n=18) |

| **Table 3 ONLINE SUPPLEMENT:** Rates of all-cause death according to heart failure at baseline during follow-up. | | | | | | |
| --- | --- | --- | --- | --- | --- | --- |
|  |  | **0-30 days** | **31-180 days** | **181-365 days** | **366-730 days** | **In total** |
| **Heart failure at baseline** | **Yes,**  n=410 | 0.7%  (n=3) | 2.2%  (n=9) | 2.4%  (n=10) | 3.4%  (n=14) | 8.8% (n=36) |
|  | **No,**  n=2561 | 0.4%  (n=9) | 0.5%  (n=13) | 0.6%  (n=14) | 1.4%  (n=36) | 3.6% (n=92) |

**Table** **4** **ONLINE SUPPLEMENT.** Adjusted survival analyses, Cox regression, hazard ratios for the composite endpoint (recurrent stroke, myocardial infarction, major bleeding, all-cause death), and all components of the combined endpoint as well as for recurrent ischemic stroke/TIA within 24 months after the index event for patients with heart failure at baseline according to LVEF < 55% on echocardiography only (n=381) compared to those without heart failure at baseline.

|  | **Crude** |  | **Adjusted, Model 1^a^** |  | **Adjusted, Model 1^a^** | |
| --- | --- | --- | --- | --- | --- | --- |
|  | **HR (95%CI)** | ***P*** | **HR (95%CI)** | ***P*** | **HR (95%CI)** | ***P*** |
| Composite endpoint^b^ | 1.55 (1.20-2.00) | 0.001 | 1.30 (0.99-1.70) | 0.056 | 1.30 (1.00-1.71) | 0.053 |
| All-cause death | 2.08 (1.40-3.09) | <0.001 | 1.61 (1.06-2.43) | 0.024 | 1.60 (1.06-2.42) | 0.025 |
| Recurrent stroke^c^ | 1.20 (0.85-1.70) | 0.302 | 1.08 (0.75-1.56) | 0.664 | 1.09 (0.76-1.57) | 0.635 |
| Recurrent ischemic stroke or TIA | 1.14 (0.79-1.64) | 0.488 | 1.06 (0.72-1.55) | 0.770 | 1.07 (0.73-1.57) | 0.736 |
| Recurrent ischemic stroke | 1.24 (0.83-1.85) | 0.299 | 1.08 (0.71-1.66) | 0.719 | 1.04 (0.67-1.63) | 0.848 |
| Recurrent TIA | 0.98 (0.47-2.01) | 0.984 | 1.08 (0.50-2.32) | 0.845 | 1.09 (0.51-2.34) | 0.827 |
| Myocardial infarction | 4.32 (2.11-8.87) | <0.001 | 2.46 (1.14-5.31) | 0.022 | 2.43 (1.12-5.27) | 0.024 |
| Major bleed | 0.61 (0.23-1.63) | 0.321 | 1.62 (0.58-4.52) | 0.355 | 1.59 (0.57-4.45) | 0.377 |

a) Adjusted Model 1: for age, stroke severity (NIHSS score on admission), diabetes mellitus, arterial hypertension, hypolipoproteinemia, coronary heart disease, detection of atrial fibrillation in-hospital, randomization; Adjusted Model 2: all variables of Adjusted Model 1 and additionally intravenous thrombolysis and endovascular thrombectomy b) Recurrent stroke, MI, major bleed, all cause death c) Ischemic Stroke, Transient Ischemic Attack, Intracerebral Hemorrhage, Subarachnoid Hemorrhage

| **Table 5 ONLINE SUPPLEMENT:** Antithrombotic treatment of MonDAFIS study patients with or without heart failure at baseline at 6, 12 and 24 months after the index stroke/TIA. | | | |
| --- | --- | --- | --- |
|  | **Heart failure**  n=410 | **No heart failure**  n=2,151 | ***p-value*** |
| **6-months follow-up** |  |  |  |
| Oral anticoagulation, n (%) | 57 (15.7) | 233 (11.8) | 0.046 |
| Antiplatelet, n (%) | 306 (84.3) | 1,660 (84.0) | 0.938 |
|  |  |  |  |
| **12-months follow-up** |  |  |  |
| Oral anticoagulation, n (%) | 57 (16.6) | 243 (12.9) | 0.072 |
| Antiplatelet, n (%) | 287 (83.4) | 1,550 (82.5) | 0.699 |
|  |  |  |  |
| **24-months follow-up** |  |  |  |
| Oral anticoagulation, n (%) | 63 (20.7) | 249 (14.1) | 0.004 |
| Antiplatelet, n (%) | 238 (78.3) | 1,407 (79.4) | 0.646 |
